# Effects of reviewing childbirth scenarios on choice of delivery type: a randomized controlled trial

**DOI:** 10.4274/tjod.galenos.2019.92260

**Published:** 2019-03-27

**Authors:** Massome Rasoli, Seyed Mohammad Mirrezaie, Ensieh Fooladi, Robabeh Zarouj Hosseini, Mahsa Fayaz

**Affiliations:** 1 Student Research Committee, School of Nursing and Midwifery, Shahroud University of Medical Sciences, Shahroud, Iran; 2 Shahroud University of Medical Sciences, Center for Health-Related Social and Behavioral Sciences Research, Shahroud, Iran; 3 Mazandaran University of Medical Sciences, School of Nursing and Midwifery, Sari, Iran; 4 Shahroud University of Medical Sciences, School of Nursing and Midwifery, Department of Medical Education, Shahroud, Iran; 5 Shahroud University of Medical Sciences, School of Public Health, Department of Epidemiology, Shahroud, Iran

**Keywords:** Cesarean section, theory of planned behavior, scenario, Iran

## Abstract

**Objective:**

The incidence of cesarean section (CS) was estimated as about 48% between 2000 and 2012 in Iran. This study was conducted to assess the effects of reviewing written childbirth scenarios on the selection of delivery method.

**Materials and Methods:**

This randomized controlled trial was conducted in Shohada Women’s Hospital in Behshahr, Mazandaran, Iran, from May to December 2015. A total of 223 women at 28 to 32 weeks of gestation were randomly allocated into three groups; the standard care (control), theory of planned behavior (TPB)-based education, and TPB education plus additional support via written childbirth scenarios (scenario). Participants were assessed at baseline (weeks 28-32) and intervention (week 37 of pregnancy) periods. Both intervention groups (TPB and scenario groups) participated in three learning sessions that were based on TPB, whereas the control group received routine care service.

**Results:**

The frequencies of normal vaginal delivery (NVD) in the scenario, TPB, and control groups were 73.2%, 58.5%, and 45.7%, respectively (p=0.004). The results showed that the relative risks of CS decision in the scenario and TPB groups in comparison with the control group were both 0.87 and statistically significant (p=0.018 and p=0.013, respectively). The relative risk of choosing CS after the removal of obligatory CS cases in the scenario group compared with the control was 0.85.

**Conclusion:**

Written childbirth scenarios that contain information on NVD and CS as additional support are effective educational tools for reducing CS rates.

**PRECIS:** Reviewing of normal vaginal delivery scenarios based on theory of planned behavior, in pregnant women could decrease relative risk of choosing CS compared with the control by 0.15.

## Introduction

According to the World Health Organization (WHO), the ideal rate of cesarean section (CS) surgeries among healthy nulliparous women is 10% to 15%, but in many countries, it has continued to increase during the past three decades^(1)^. For example, CS rates in Latin America and the Caribbean, Europe, Asia, and Iran are 40.5%, 25%, 19.2%, and 48%, respectively^(2,3)^.

Among pregnant Iranian women, 70% of those who opt for CS delivery do so for reasons other than medical needs, including personal request, spouse’s priorities, and sometimes, inducement by physicians, so the mother’s decision is one of the main factors for the increase in CS deliveries^(4)^. Evidence suggests that training women to develop skills of overcoming a fear of normal vaginal delivery (NVD), reducing labor pain^(5)^, raising women’s awareness regarding the advantages and disadvantages of a given delivery method^(6,7)^, and engaging them in the process of decision-making on delivery method^(8)^ are effective ways of reducing CS-favoring decisions. The effectiveness of health education programs is substantially related to applied educational theories and models^(9)^. One of the most important theories for predicting and understanding behavior is the theory of planned behavior (TPB), whose effectiveness has been confirmed in experimental studies^(9,10)^. The theory maintains that a particular behavior can be predicted on the basis of behavioral intention. Based on the theory of planned behavior, if a person assumes a behavior to be reflective of good conduct (positive attitude), they believe that others will regard it as valuable behavior (positive mental norm); accordingly, they will also intend to perform it.^(11)^

Childbirth stories or scenarios help women to select the best delivery mode because these narratives help them identify unknown aspects of delivery, reduce fear, and increase a sense of control^(12)^. Various studies have confirmed the importance of scenarios in learning^(13,14)^. Peer experiences, such as narrating childbirth stories, influence the choice of delivery method^(15)^ because negative stories about NVD motivated pregnant women to select CS^(16,17)^. In a study conducted by Blainey and Slade (2015), the writing and sharing of childbirth stories by women who experienced traumatic delivery exerted positive mental health effects on the women^(18)^.

To address this capability of scenario, the present study evaluated the effects of reviewing written childbirth scenarios on the decision of nulliparous women regarding delivery method (i.e., NVD vs. CS). The examination was based on TPB.

## Materials and Methods

### Study design

The study was designed as a parallel randomized controlled trial with three arms conducted in the prenatal clinic of the gynecology department of a governmental hospital, Shohada Women’s Hospital of Behshahr, a northern Iranian city located in the Mazandaran province. The study received an ethics code (Ir.shmu.rec.1394.32) from Shahroud University of Medical Sciences, and was approved by Mazandaran University of Medical Sciences. The study was registered in the Iranian Center for Registration of Clinical Trials (IRCT2015052020706N2) and the study protocol is available in http://fa.search.irct.ir/search?query=IRCT2015052020706N2.

### Participants

All pregnant women who visited the clinic from May through December 2015 were considered for the study. To recruit suitable participants, we approached eligible women during their first visit to the clinic. The inclusion criteria were primigravid pregnant women, single-fetus pregnancies, gestational age of 28 to 32 weeks, maternal age of 18 to 35 years, no history of frequent abortion, and no contraindication for normal childbirth. The exclusion criteria were unwillingness to participate in training courses, absence from one of the training sessions, and obstetric or medical contraindications to vaginal birth and/or trial of vaginal birth (e.g., placenta previa). All participants gave written informed consent.

### Randomization and masking

**Figure 1:**
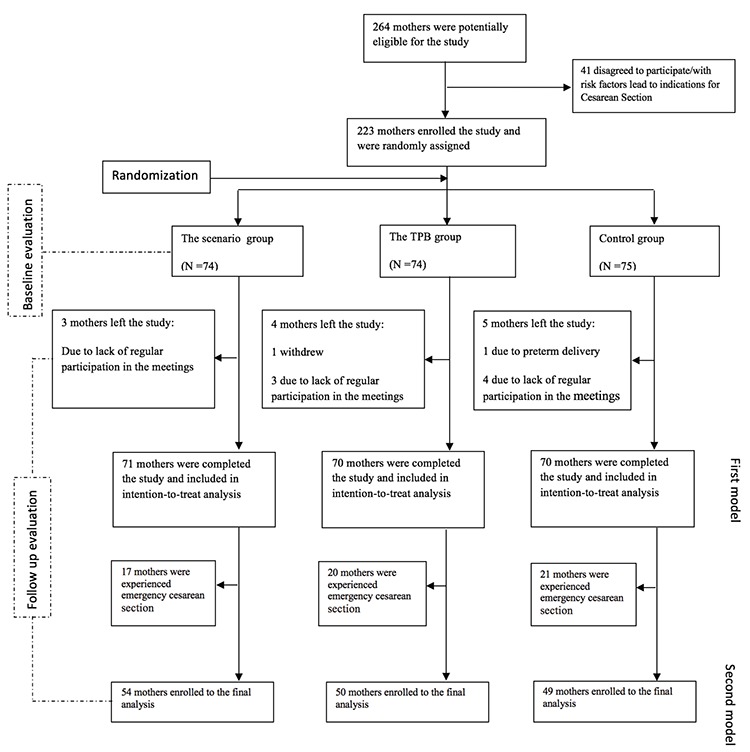
Trial profile TPB: Theory of planned behavior

Using computer-generated random blocks of six, we randomly assigned 233 eligible participants to three study groups with an allocation ratio of 1:1, namely, two intervention groups [TPB-based education (TPB group) and TPB-based education plus additional support via written childbirth scenarios (scenario group)], each consisting of 74 participants, and a control group, who received standard care (75 participants) (Figure 1). The randomization was supervised by statisticians who were not involved in the enrolment or follow-up of participants. The clinic’s employees could not be subjected to masking from the group assignments because of the nature of the interventions.

### Procedures

For the implementation of TPB-based education and TPB-based education plus additional support with written childbirth scenarios, we trained the healthcare staff of the prenatal and maternity departments of the intervention facility by using a WHO course^(18)^. Considering the lack of a standard questionnaire in this field, a study-specific instrument was developed on the basis of TPB resources and textbooks and the results of previous studies^(19,20,21)^. The questionnaire comprised 80 questions distributed across two sections. The first section, whose composition was based on the questions used by Hildingsson et al.,^(20)^ revolves around awareness related to childbirth, socio-demographic characteristics, and obstetric background. The specific items falling under this section are those related to demographics (age, occupation, and education of the expectant mother and her spouse, place of residence, and economic status), midwifery issues (e.g., date of last menstrual period, probable date of delivery, gestational age, gravity, parity, number of embryos in the current pregnancy), awareness (10 items on awareness about normal childbirth and CS, pros and cons of different delivery methods for the mother and the fetus, and scientific indications of CS), and results evaluation (10 items). To assess the economic situation, the questionnaire also presents questions regarding household assets. Using principal components analysis and considering three layers, we classified economic status into high, medium, and low levels. The second section of the questionnaire consists of the end-structures of TPB, including attitude toward an act (16 items), subjective norms (5 items), perceived behavioral control (3 items), behavioral intention (2 items), and behavioral performance (1 item).

Pregnant women belonging to the intervention groups were scheduled for educational TPB-based interventions in groups of 10 to 12 people; however, the control group was administered only routine services by the healthcare staff. The educational interventions were conducted at three 60-minute weekly sessions. The structures of the sessions are presented in Table 1 (see appendix 1 for more details regarding the training). Both the interventional groups and the control group received a training manual on the advantages of NVD and the non-pharmacologic methods of pain relief.

The written scenarios, which were composed on the basis of actual situations, consisted of six positive stories about the physiological childbirth process with non-pharmacologic pain relief methods that were helpful to mothers and two negative stories about unscientific CS deliveries that were performed at the mothers’ request (appendix 2). The scenario group was asked to read the scenarios before the next meeting and identify the points that they believed were useful, interesting, and functional.

At each subsequent session, the stories were reviewed with the participants to help them improve their understanding of difficult sentences, increase the attractiveness of the stories, and create a deep connection to the narratives. The practical strategies for story review included pausing, thinking, and retelling. Encouraging individuals to pause after reading part of a story, ponder over this part, and retell it to others helps them better learn about the story; it also allows them to determine how much of the story they can recall^(22)^. After a review of each scenario, the participants were asked to express their feelings and impressions of each scenario.

The participants of the three groups were asked to complete a pre and post-test questionnaire at 37 weeks of pregnancy and one month after the completion of the educational interventions, respectively.

### Outcomes

We assessed one primary outcome, that is, the proportion of mothers who intended to opt for NVD, within one month after the completion of the educational interventions. This decision was based on TPB’s argument that intention is the main factor that determines behavior (see Introduction). We also assessed a secondary outcome, that is, the prevalence of NVD selection among the mothers.

### Statistical Analysis

Mean ± SD (standard deviation) and frequency were reported as descriptive statistics. ANOVA was performed to compare the continuous outcomes of the groups. Chi-square and Fisher’s exact tests were also performed for categorical variables. Log-binomial regression models were used to explore the relative risk (RR) of the decision to undergo a CS (first model) and the risk of CS delivery (second model) in the groups for which other covariates were adjusted. In the modeling, the control group was considered as a reference category. All the analyses were conducted in STATA version 12, and the significance level was considered as 0.05.

## Results

As previously stated, we randomly assigned 223 participating mothers to three groups; 74, 74, and 75 women were assigned to the scenario (PTB + written scenarios), PTB (PTB-based education only), and control (routine care) groups, respectively. A total of 211 participants were included in the analysis. The loss to follow-up rate was about 5%. The mean age of the women was 24.9±4 years. Some of them had diplomas (34.6%), and many were housewives (88.6%). The baseline characteristics of the women according to groups are shown in Table 2.

The primary decision for delivery chosen by the study groups at the beginning of the study showed no significant differences (Table 2). However, the frequencies of NVD selection in the scenario, TPB, and control groups as a primary outcome were 73.2%, 58.5%, and 45.7%, respectively (p=0.004).

In the first log-binomial regression model, the regression was run on the decision for CS as an outcome variable in the presence of other covariates. The results showed that the RR of the decision to undergo CS in the scenario and TPB groups in comparison with the control group were both 0.87 and statistically significant (p=0.018 and p=0.013, respectively). The findings also indicated that the primary decision to undergo CS (RR=1.26, p<0.001) and a high level of education (RR=1.04, p=0.004) were associated with an increased RR of the decision to undergo CS. An increase in perceived behavioral control and in awareness scores decreased the RR of the decision for CS (Table 3).

The number of CS deliveries performed in this study was 86 (40.76%), among which 58 (67.44%) and 28 (32.56%) were performed for emergency and elective reasons, respectively. In the second model, women for whom obligatory CS delivery was prescribed were excluded, leaving us with a sample of 153 participants. The log-binomial regression with CS as an outcome variable in the presence of covariates was repeated. The findings showed that the RR of CS in the scenario group was 0.85, which was statistically significant (p=0.005). No statistical difference was found between the TPB and control groups. The primary decision to opt for CS (RR=1.18, p<0.001) and higher level of education (RR=1.06, p=0.001) increased the RR of CS. Increased in the difference of perceived behavioral control score between pre and post-test was decreased RR of CS decision (RR=0.97. p=0.004) (Table 3).

## Discussion

Our results showed that using positive stories about childbirth in the TPB model is an effective strategy for enhancing the predilection of pregnant women to select NVD as a delivery mode. In both intervention groups, TPB strongly predicted behavioral intention. The interventions were also associated with a significantly reduced prevalence of CS decision in the intervention groups. Although the written scenarios exerted the greatest effects on the behavioral constructs, no significant difference in behavioral performance was found between the TPB and control groups.

In a study conducted in northern Iran, Besharati et al.^(19)^ found that intervention based on TPB effectively reduced the incidence of elective CS and increased the selection of NVD. However, only half of the participants of intervention group who exhibited an intention to select NVD actually chose this type of delivery.

In the current research, after the removal of emergency CS cases, the log-binomial regression showed that the RR of CS decision significantly decreased in the scenario group compared with the risk observed in the control group (p=0.005). The RR of CS decision in this group relative to the control and TPB groups decreased by 0.15 and 0.07, respectively. In a study performed by Regan et al.,^(23)^ 71.2% of the women stated that the most important and valuable resource that helped them to select a method of delivery was stories of childbirth by other women. 

In our study, stories as powerful educational tools had a significant effect on the behavioral tendency of the nulliparous women to reduce CS decisions. This effect seems to have originated from the characteristics of the stories, which are believable and memorable. They are believable because they originated from human and pseudo-human experiences that people are inclined to perceive as authentic scientific sources. They are memorable because they inspire involvement in the characters’ intentions and performance^(24)^. Bruner (1986) argued that stories developed an awareness and performance outlook in humans and that these two were part of human intentions; an audience becomes involved with a story at both levels, and through this involvement, enters the minds of characters and achieves an improved understanding of story meanings^(25)^.

Hearing negative stories about normal childbirth was one of the factors that triggered the decision to opt for CS among women in a study conducted in northern Iran^(16)^. In a study performed in North Carolina, Romero et al.^ (26)^ discovered that the formation of the initial fear of normal childbirth in some women was due to the fact that they heard unfavorable stories from friends and acquaintances. On the other hand, confidence in one’s ability to have NVD and the belief that vaginal childbirth is a normal method of giving birth were among the principal drivers of NVD decision.

Our study showed that the intention to opt for vaginal delivery in the two intervention groups significantly differed from that in the control group. After the educational interventions, the changes in women’s awareness and perceived behavioral control were associated with a reduced risk of CS decision. Additionally, a direct correlation was found between the initial decision of women to undergo CS and their levels of education and the risk of CS decision. Rahman et al.^(27)^ (2014) and Rajabi et al.^ (28)^ (2015) in Iran and in Malaysia found that high levels of education among mothers were associated with an increase in CS delivery.

In the scenario group, projection with the heroines in the stories helped the pregnant women recognize their latent capacities and eliminate their unknown fears and concerns. Lindesmith and McWeeny^(29)^ found that a story established a connection between women and their shared backgrounds and that sharing stories regarding childbirth, as a seminal experience, should officially be taken into consideration in childbirth training programs. In a review, Webb et al.^(30)^ (2010) found that interventions based on TPB had a significant effect on behavior. The authors emphasized that the closer the integration of intervention with behavioral change techniques, the better the performance effects derived.

In the NVD scenarios, the heroine-like roles of the characters and confidence in women’s power to manage labor pains improved the perceived behavioral control capabilities of the readers. For these women, therefore, normal childbirth was viewed as achievable and realistic. A study showed that nulliparous women regarded childbirth stories as a way of facilitating the selection of normal childbirth. The study also indicated that creating new positive real stories or narratives effectively reduced the choice of CS delivery and increased the motivation to select normal childbirth^(31)^. In the current study, the infrastructures of the stories helped instill the truth in the mindsets of the women and improve the decision-making process underlying childbirth.

To the best of our knowledge, although childbirth stories are often used in qualitative research, in the present study we used them in a clinical trial to determine their efficacy. A limitation worth mentioning is that the effects of traumatic delivery scenarios on the choice of delivery method were not examined, also the study involved nulliparous women only.

This present study shows that a combination of childbirth scenarios and training based on TPB could be used to reduce the incidence of unnecessary CS deliveries.

## Figures and Tables

**Table 1:**
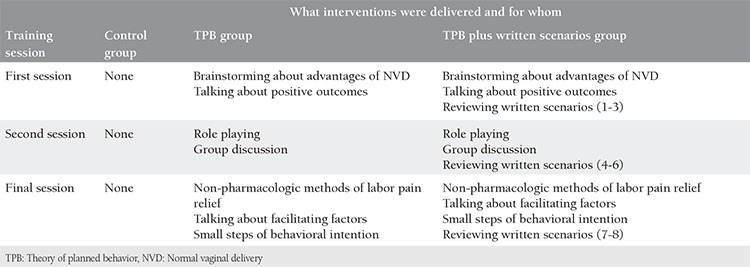
Intervention by study groups

**Table 2:**
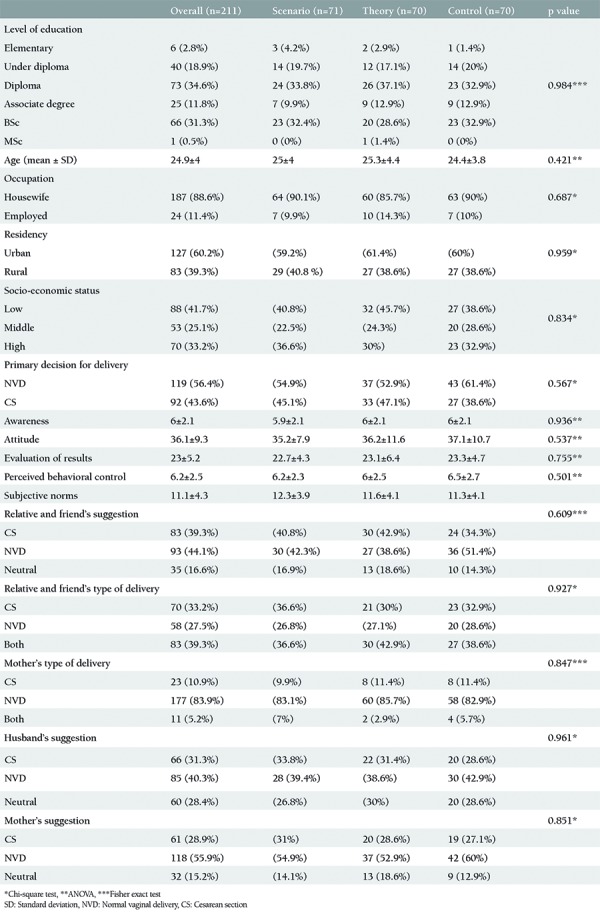
Baseline characteristics of participants

**Table 3:**
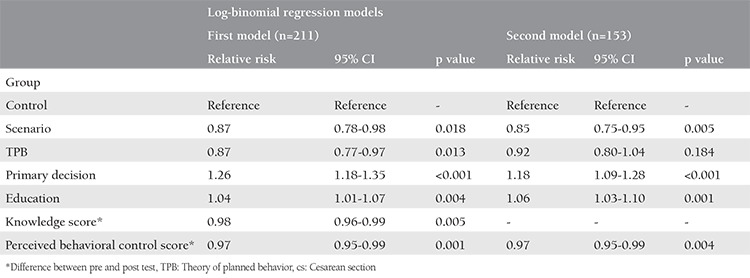
Log-binomial regression on CS decision (first model) and CS delivery (second model)
